# The effects of chronic betaine supplementation on body composition and performance in collegiate females: a double-blind, randomized, placebo controlled trial

**DOI:** 10.1186/s12970-018-0243-x

**Published:** 2018-07-31

**Authors:** Jason Michael Cholewa, Andrea Hudson, Taylor Cicholski, Amanda Cervenka, Karley Barreno, Kayla Broom, McKenzie Barch, Stuart A. S. Craig

**Affiliations:** 1 Department of Kinesiology, Coastal Carolina University, PO Box 261954, Williams-Brice 101A, Conway, SC 29528 USA; 2 Regulatory & Scientific Affairs, DuPont Nutrition & Health Experimental Station, Wilmington, DE USA

**Keywords:** Aesthetics, Periodization, Hypertrophy, Resistance training, Fat loss, Ergogenic aid

## Abstract

**Background:**

Betaine supplementation has been shown to improve body composition and some metrics of muscular performance in young men; but, whether betaine enhances body composition or performance in female subjects is currently unknown. Therefore, the purpose of this study was to investigate the interaction between resistance training adaptation and chronic betaine supplementation in females.

**Methods:**

Twenty-three young women (21.0 ± 1.4 years, 165.9 ± 6.4 cm, 68.6 ± 11.8 kg) without prior structured resistance training experience volunteered for this study. Body composition (BodPod), rectus femoris muscle thickness (B-mode Ultrasound), vertical jump, back squat 1RM and bench press 1RM were assessed pre- and post-training. Following 1 week of familiarization training, subjects were matched for body composition and squat strength, and randomly assigned to either a betaine (2.5 g/day; *n* = 11) or placebo (*n* = 12) group that completed 3 sets of 6–7 exercises per day performed to momentary muscular failure. Training was divided into two lower and one upper body training sessions per week performed on non-consecutive days for 8 weeks, and weekly volume load was used to analyze work capacity.

**Results:**

Significant main effects of time were found for changes in lean mass (2.4 ± 1.8 kg), muscle thickness (0.13 ± 0.08 cm), vertical jump (1.8 ± 1.6 cm), squat 1RM (39.8 ± 14.0 kg), and bench press 1 RM (9.1 ± 7.3 kg); however, there were no significant interactions. A trend (*p* = .056) was found for greater weekly training volumes for betaine versus placebo. Significant interactions were found for changes in body fat percentage and fat mass: body fat percentage and fat mass decreased significantly more in betaine (− 3.3 ± 1.7%; − 2.0 ± 1.1 kg) compared to placebo (− 1.7 ± 1.6%; − 0.8 ± 1.3 kg), respectively.

**Conclusions:**

The results of this study indicated that betaine supplementation may enhance reductions in fat mass, but not absolute strength, that accompany a resistance training program in untrained collegiate females.

## Background

Betaine anhydrous (trimethylglycine) is a naturally occurring byproduct of sugar beet refinement that may improve changes in body composition and muscular performance during a resistance training protocol [1]. Betaine is high in other foods such as wheat bran, wheat germ, spinach, beets, and wheat bread, although exact values will vary highly with different sources and cooking methods [2]. The average betaine intakes in adult humans are approximately 100–400 mg/day [3], however the ergogenic and clinical effects of betaine have been investigated with doses ranging from 500 to 20,000 mg/day [4–8].

Betaine in dosages of 2.5 g/day for 14 days to 6 weeks has shown potential to enhance strength-based performance [1]. Hoffman et al. [9] demonstrated improved squat repetitions to fatigue, but not bench press throw or vertical jump with 15 days of 2.5 g/day betaine supplementation; however, a later study by Hoffman et al. [10] reported no improvements in isokinetic force output following an identical supplementation protocol. In contrast, Lee et al. [11] demonstrated improvements in vertical jump power output, bench press throw power output, and force production in the isometric back squat and bench press with 12 days of betaine supplementation. Our lab [7] demonstrated improvements in bench press, but not back squat, training volume and a trend for improved vertical jump with 6 weeks of betaine supplementation. Unlike Hoffman et al. [10], the subjects in Cholewa et al. [7] and Lee et al. [11] were assigned standardized resistance training between testing sessions. Therefore, these studies provide evidence that indicates betaine supplementation may be effective in improving muscular performance when accompanied by structured resistance training.

The effects of betaine supplementation on body composition and hypertrophy in humans is limited. Schwab et al. [8] reported 12 weeks of 6 g/day betaine supplementation did not improve body composition or resting energy expenditure in obese, sedentary subjects. Del Favero et al. [12] reported similar findings with 2 g/day betaine supplementation for 10 days in untrained young men who were instructed not to exercise. To our knowledge only one study has investigated the interactions between betaine supplementation and resistance training adaptation [7]. In that study, experienced resistance trained men were divided into two groups, prescribed a progressive resistance training program, and supplemented with 2.5 g/day betaine for 6 weeks. Compared with placebo, betaine increased lean mass, reduced fat mass, and increased arm, but not leg, lean cross sectional area [7]. Although numerous studies show improvements in body composition in animals without an exercise component [13], caution should be taken when translating these results to humans as livestock studies most commonly utilize animals still in the development phase whereby the growth of long bones places a stress upon the musculature that is absent in adults. The limited body of previous research indicates that betaine supplementation when performed in conjunction with resistance training seems to result in improvements in body composition, however, more research is necessary to verify this hypothesis.

The mechanisms by which betaine affects strength and body composition are still not fully understood. By acting as a methyl donor, betaine may increase creatine availability [9] or enhance the protein kinase B–mechanistic target of rapamycin (Akt-mTOR) pathway [14]. Betaine that does not participate in methylation metabolism is readily taken up by tissues and used as an organic osmolyte in the regulation of cell volume [15]. External osmotic stress increases the cellular accumulation osmolytes, and the osmotic stress of exercise may increase betaine uptake by skeletal muscle. As an osmolyte, betaine has been shown to stabilize cellular metabolic functions [16], enhance protein stability under osmotic stressors [17], and protect myosin ATPase and myosin heavy chain proteins against denaturation by urea [18]. Females present with lower plasma betaine concentrations than males, possibly as a result of higher betaine catabolism due to faster rates of methylation metabolism [19]; however, it is currently unknown if females respond differently to betaine supplementation than males.

To date, only one study has evaluated the effects of betaine supplementation in females. This study was shorter in duration (7 days) and did not evaluate the interaction between betaine supplementation and training, nor did it measure body composition [20]. The purpose of this study is to evaluate the effects of 9 weeks of betaine supplementation with resistance training on performance and body composition in young, active females. We hypothesized that betaine would increase lean mass, reduce fat mass, and improve strength performance.

## Methods

### Experimental design

The present study was designed to evaluate the effects of 9 weeks of betaine supplementation and resistance training on body composition and physical performance in active, resistance training naïve, young women. A double-blind independent groups design was used. Body composition, body water, and physical performance were measured pre- and post-treatment. All methods and procedures were approved by the University Institutional Review Board (IRB #2016.52) and written and signed informed consents were obtained from all subjects prior to data collection.

### Subjects

Subjects were a convenience sample of female volunteers recruited from a university population. Subjects were between the ages of 18–35, without any existing musculoskeletal disorders, and free from consumption of anabolic steroids or any other illegal agents known to increase muscle size during the previous year. In this study, active resistance training naïve females were defined as subjects who were engaged in moderate intensity aerobic exercise, not competing in sports (i.e.: not on an NCAA or club sports team) and had not performed any regimented resistance training for the past 6 months. Subjects were encouraged to maintain their current level of activity in addition to the supervised resistance training program and were instructed not to adopt a new exercise program during the study. Subjects were instructed to avoid taking any performance-enhancing supplements (i.e.: beta alanine, creatine, fat burners, and pre-workout supplements) during the study period. A minimal adherence of 92% (completion of 22 of 24 total training sessions) was set a priori and make up sessions were made available to subjects when possible. Subjects that missed a total of 3 training sessions or that missed two training sessions in a row were disqualified from the study.

Because changes in body fat percentage were a primary outcome, in this study we performed an a priori power calculation based on body fat percentage from a previous betaine study we performed in men [7]. Based upon the delta differences in body fat percentage, with an alpha of 0.05 and beta of 0.80, we calculated we would need at least 9 subjects per group. Given a previous dropout rate of 33% in the population being studied [21], we calculated we would need to recruit at least 15 subjects per group.

A total of 38 subjects qualified for the study, however 2 dropped out prior to group placement, and therefore 36 subjects were divided into a treatment and placebo group. To form groups, subjects were assigned an identification number and then placed into one of the six following categories: “strong lean”, “weak lean”, “strong normal”, “weak normal”, “strong fat” and “weak fat”, whereby strong corresponded to a squat 1 RM > 60 kg and weak < 60 kg, and body fat percentages of < 23%, 25–35%, > 35% corresponded to “lean”, “normal”, and “fat”, respectively. The median baseline squat was 60.22 kg and the body fat percentage values corresponded to the approximate tertials of the range. A colleague not involved in the study then randomly assigned subjects via their identification number to either a treatment or placebo group, ensuring that each group was equally represented by each category. The blind was not removed until all data had been collected and analyzed.

### Procedures

**Fig. 1:**
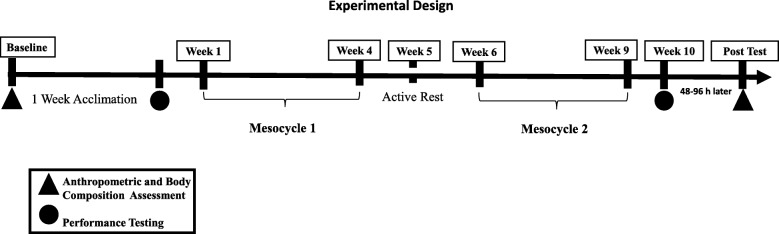
Experimental Design Timeline

Testing was conducted in the following order: anthropometrics, power, and strength testing. For baseline testing, anthropometrics were measured on a separate day prior to any training, and power and strength testing was conducted following a week of familiarization training (Fig. 1). Subjects performed 3 familiarization sessions (Monday, Wednesday, and Friday) separated by 48 h prior to the power and strength testing which occurred the following Monday. Resistance training then commenced on Wednesday. Subjects were asked not to alter their diets. Dietary intake was measured pre-treatment and post-treatment via 3-day food logs that consisted of 2 weekdays and 1 weekend day to better reflect typical intakes. Subjects met with the primary investigator and were instructed how to complete the food logs. Subjects were asked to replicate their pre-treatment nutritional intakes the days of post-treatment strength and performance testing to reduce the influence of nutritional status affecting the results. Total energy intake, carbohydrate, protein, and fats was measured pre- and post-treatment via Diet Analysis Plus Version 10 (Cengage, USA) and converted into relative values (kcal or grams per kg of body mass) to compare intakes across time and between groups.

### Anthropometrics

#### Body composition

Height was measured to the nearest 0.1 cm without shoes using a stadiometer. Body mass was measured to the nearest 0.1 kg using an electronic scale (Cosmed, Concord, CA USA). Body composition was measured pre- and post-treatment and was determined by whole body densitometry using air displacement plethysmography (Bod Pod®, Cosmed, Concord, CA USA). All testing was performed in accordance with the manufacturer’s instructions and subjects were tested while wearing only tight-fitting compression shorts, sports bra, and Lycra swim cap. Subjects were instructed to wear the same clothing for all testing procedures, to not consume food or drink 3 h prior to testing, and to consume a similar quantity of food on both sessions. All testing was carried out at approximately the same time of day (± 1 h) to account for circadian changes in fluid and fecal matter. Data from the Bod Pod® included body weight, percent body fat, fat free mass and fat mass. Based upon a small pilot study (*n* = 6), the ICC and SEM for percent body fat from our lab are 0.998 and 0.56%, respectively.

#### Measurement of compartmental water

Total body water (TBW), intracellular water (ICW), and extracellular water (ECW) was assessed using the Quantum IV bioelectrical impedance analyzer and accompanying software (BIA: RJL Systems, Clinton Township, MI) in accordance with procedures described elsewhere [22]. Bioelectrical impedance spectroscopy has been shown to be a valid tool for assessment of TBW and its various compartments in young women [23–27]. Prior to measurement, urine specific gravity was assessed and subjects with a urine specific gravity greater than 1.025 were asked to sip water and return an hour later. A small pilot study (*n* = 7) with college aged females was conducted and *Chronbach’s alpha* test–retest reliability and the standard error of measurement were α = 0.97 and 3.0 L, respectively, for total body water.

#### Muscle thickness

Muscle thickness of the right rectus femoris was obtained via a B-mode ultrasound imaging unit (ECO3; Chison Medican Imaging, Jiang Su, China). A 5 MHz ultrasound probe with water-soluble transmission gel was placed on the measurement site perpendicular to the tissue interface without depressing the skin. When the quality of the image was satisfactory it was saved to a hard drive and muscle thickness dimensions were obtained by measuring the distance from the subcutaneous adipose tissue-muscle interface to the deep aponeurosis according the Abe et al. [28] protocol. Measurements were taken at 50% distal between the anterior superior iliac spine and proximal border of the patella. To control for any effects of cellular swelling in the muscles due to training, all images were obtained 72–96 h following the final training session [29]. To reduce test-retest variability, the same tester obtained all images and measurements. Based on a small pilot study (*n* = 7), the ICC and SEM for rectus femoris muscle thickness from out lab are 0.984 and 0.03 cm, respectively.

### Physical performance testing

Physical performance testing occurred in the following order: vertical jump, back squat 1 repetition maximum (RM), and bench press 1 RM. A standardized 5 min aerobic warmup was performed prior to the first performance test.

#### Lower body power

Vertical jump was assessed using the Just Jump! Mat (Probotics Inc.: Huntsville, AL). Leard et al. [30] demonstrated that the Just Jump! Mat is highly correlated (*r* = .97) with the 3-camera video analysis “gold standard” method of assessing vertical jump performance. Subjects were instructed to stand on the mat with feet hip-width apart and perform a rapid lower body eccentric contraction followed immediately by a maximal intensity concentric contraction. The best of the three trials was recorded in cm as vertical jump height. Based upon a small pilot study (*n* = 6), the ICC and SEM from our lab are 0.991 and 1.50 cm, respectively.

#### Muscle strength

Following a 3 min rest subjects’ 1RM squat and bench press were tested. Repetition maximum testing was consistent with recognized guidelines as established by the National Strength and Conditioning Association [31]. Subjects were required to reach parallel (iliotibial band parallel to the floor) in the 1RM squat for the attempt to be considered successful as determined by the primary investigator. Subjects were required to touch the bar to their lower chest without bouncing for the 1RM bench press to be considered valid. All 1RM determinations were made within 5 attempts and a 5 min passive rest separated the squat testing from the bench press testing. Based on results of a small pilot study (*n* = 5), the test-retest ICC and SEM from our lab for back squat 1RM testing was 0.961 and 2.37 kg, respectively. For bench press 1RM pilot testing (*n* = 6) revealed an ICC and SEM of 0.980 and 1.00 kg, respectively.

### Resistance training protocol

Subjects assigned to the betaine and placebo groups performed the same exercises, sets, and repetitions during the investigation. Training consisted of 3 weekly sessions performed on non-consecutive days for a total of 10 weeks and all training sessions were supervised. The first 3 sessions (week 1) were an acclimation phase where sets were performed with 2–3 repetitions in reserve [32] and with a repetition range of 8–12. For the final 9 weeks all sets were performed until momentary concentric muscular failure (Fig. 1). A 9 week training period was selected since several similar studies have demonstrated changes in muscle growth and body composition following 8 weeks of resistance training [33–35]. The inclusion of two lower body and only one upper body sessions was selected because we measured rectus femoris thickness and our previous research demonstrated greater increases in thigh compared to arm cross sectional area with twice weekly lower body training [21]. Since all subjects entered the study at the same time, week 5 corresponded with spring break and provided subjects with a week of active rest between the transitions from mesocycle 1 to mesocycle 2. Repetitions were controlled with a cadence of approximately 1 and 2 sec for concentric and eccentric actions, respectively. Subjects were afforded 2 min rest between sets of bilateral multi joint movements and 1 min of rest between ancillary movement sets. The starting load for the bench press and squat exercise corresponded to 65% of the 1 RM. The load was adjusted for each exercise on an as needed basis to ensure that subjects achieved failure in the target repetition range, and attempts were made to progressively increase the load on a weekly basis. Training loads from each session were recorded and weekly volume was calculated as the sum of the load lifted multiplied by the number of repetitions performed. The training protocol can be found in Table 1.

**Table 1:** Resistance training protocol

Mesocycle 1 (Weeks 1–4)		Mesocycle 2 (Weeks 5–9)	
Exercise	Rx	Exercise	Rx
Monday			
Squat	3 × 10–12	Squat	3 × 8–10
Rumanian Deadlift	3 × 10–12	Rumanian Deadlift	3 × 8–10
Traveling Lunge	3 × 10–12	Split Squat	3 × 10–12
Leg Extensions	3 × 10–12	Leg Extensions	3 × 10–12
Lying Leg Raises	3 × 15	Lying Leg Raises	3 × 15
Russian Twists	3 × 16	Russian Twists	3 × 16
Wednesday			
Bench Press	3 × 10–12	Bench Press	3 × 8–10
DB Row	3 × 10–12	DB Row	3 × 8–10
Overhead Press	3 × 10–12	Overhead Press	3 × 10–12
Lat Pull Downs	3 × 10–12	Lat Pull Downs	3 × 10–12
DB Curls	3 × 10	Hammer DB Curls	3 × 10
Triceps Press Down	3 × 10	OH DB Tri Extension	3 × 10
Friday			
Squat	3 × 10–12	Squat	3 × 8–10
Leg Press	3 × 10–12	Leg Press	3 × 8–10
Hip Thrust	3 × 10–12	Reverse Lunge	3 × 10–12
Leg Extensions	3 × 10–12	Leg Extensions	3 × 10–12
Crunches	3 × 15	Crunches	3 × 15
Russian Twists	3 × 30 s	Russian Twists	3 × 30 s

### Treatments

Treatments were administered double-blind via pre-filled gelatin capsules and consisted of either a placebo (sugar, approximately 0.75 g/capsule) or betaine (BetaPower®, Finnfeeds Oy, Finland). The blind was not removed until all data had been collected. Subjects consumed 2 capsules (0.625 g/capsule) twice per day yielding an absolute total of 2.5 g betaine. This dosage was chosen because: betaine is safe at a dietary intake of 9–12 g/day [15]; 2.5–5 g betaine has been shown to significantly elevate plasma betaine [8, 36]; 2.5 g positively affects strength performance and body composition [1]; and the average relative dosage on the basis of lean body mass (LBM) (~ 40 mg/kg-LBM) in the present study was similar to the average relative dosage (36.3 mg/kg-LBM) reported by Hoffman et al. [9] to improve performance.

### Statistical analysis

All data is reported as means ± standard deviations. Pre-intervention differences in body composition and strength were assessed using independent samples *t*-tests. A 2 × 8 mixed factorial analysis of variance (ANOVA) with repeated measures (group x time) was used to compare weekly training volumes. A 2 × 2 (group x time) mixed factorial ANOVA with repeated measures was used to compare changes in dietary intakes, body composition, compartmental water, and performance between groups. When a significant main effect of group or interaction was found relative percent differences were calculated (percent difference = ([post-intervention measure – baseline measure] / baseline measure) × 100) and compared with independent samples t-tests with the Bonferroni correction. The normality of the data was checked and subsequently confirmed with the Shapiro-Wilk test. For all measured variables, the estimated sphericity was verified according to Mauchly’s W test, and the Greenhouse–Geisser correction was used when necessary. Effect sizes were defined as small, medium, and large and are represented by Cohen’s d of greater than 0.2, 0.5, and 0.8, respectively. All analyses was completed using SPSS Version 22 (IBM, USA) and an alpha level of *p* < .05 was set a priori.

## Results

Twenty three subjects (Table 2; betaine: *n* = 11; placebo: *n* = 12) completed the study and were included in the final analysis: one dropped out as a result of an injury that occurred outside of training, three were disqualified for missing training sessions, two dropped out due to illnesses not associated with the study procedures (mononucleosis), and the remaining seven dropped out due to personal or non-disclosed reasons. There were no significant (*p* > .05) differences between groups at baseline for any of the dependent variables. Training adherence in the treatment group was 98.1 ± 3.4%: two subjects missed two sessions and one subject missed one session. Adherence in the placebo group was 97.2 ± 3.2%: two subjects missed two sessions and four subjects missed one session. The supplement was well tolerated as zero subjects dropped out as a result of supplementation and no subjects reported any side effects.

**Table 2:** Subject baseline characteristics^*^

	Betaine (*n* = 11)	Placebo (*n* = 12)
Age (years)	20.7 ± 1.4	21.2 ± 1.3
Height (cm)	167.0 ± 5.5	164.9 ± 7.2
Body Mass (kg)	70.2 ± 13.8	67.1 ± 10.1
Body Fat Percentage	33.1 ± 9.3	32.3 ± 6.2
1 RM Squat (kg)	63.2 ± 17.9	57.2 ± 15.2
Relative Squat (1RM/kg body mass)	0.90 ± 0.18	0.85 ± 0.16

^*^No significant differences between groups for any variables (*p* > .05)

### Body composition and muscle growth

Body composition, compartmental water, and rectus femoris thickness data are displayed in Table 3. Body mass increased (*p* = .016, *F* = 6.89) similarly in both groups (*p* = .522, *F* = 0.42). Body fat percentage decreased (*p* < .001, *F* = 50.06), with a significant difference between groups at post-testing (*p* = 0.038, *F* = 4.90). Post hoc analysis revealed a significantly greater (*p* = .038, *t* = − 2.21) decrease in body fat percentage for betaine (− 3.3 ± 1.9%) compared to placebo (− 1.7 ± 1.6). Fat mass decreased (*p* < .001, *F* = 31.90) with a significant difference between groups at post-testing (*p* = 0.020, *F* = 6.33). Post hoc analysis revealed a significantly greater (*p* = .018, *t* = − 2.55) relative decrease in fat mass for betaine (− 2.0 ± 1.1 kg) compared to placebo (− 0.8 ± 1.3 kg). Fat free mass increased (*p* < .001, *F* = 42.00) similarly in both groups (*p* = .194, *F* = 1.80). Total body water increased (*p* = .005, *F* = 9.68) similarly between groups (*p* = .327, *F* = 1.82). Intracellular water increased (*p* = .005, *F* = 9.53) similarly between groups (*p* = .504, *F* = 0.46). Extracellular water was not different between pre- and post-testing (*p* = .140, *F* = 2.35), nor were there any differences (*p* = .205, *F* = 1.70) in urine specific gravity. In regards to muscle growth, rectus femoris muscle thickness increased (*p* < .001, *F* = 73.20) similarly in both groups (*p* = .976, *F* = 0.01).

**Table 3:** Body composition outcomes

		Pre-Training	Post-Training	Effect Size	95% CI for Δ
Body Mass (kg)	B	70.2 ± 13.8	70.9 ± 13.8 ^a^	0.06	−0.55, 1.93
	PL	67.1 ± 10.1	68.3 ± 10.6 ^a^	0.10	0.18, 2.13
Body Fat (%)	B	33.1 ± 9.3	29.7 ± 9.5 ^ab^	− 0.44	−4.58, − 2.08
	PL	32.3 ± 6.2	30.6 ± 5.8 ^a^	− 0.23	− 2.74, − 0.74
Fat Mass (kg)	B	23.9 ± 11.2	21.9 ± 11.4 ^ab^	− 0.22	−2.76, − 1.30
	PL	22.0 ± 6.5	21.3 ± 6.6 ^a^	− 0.09	− 1.59, 0.03
Fat Free Mass (kg)	B	46.0 ± 6.8	48.9 ± 6.3 ^a^	0.49	1.31, 4.56
	PL	45.1 ± 5.6	47.0 ± 5.6 ^a^	0.32	1.33, 2.52
Total Water (L)	B	32.8 ± 4.4	33.7 ± 4.3 ^a^	0.22	−0.78, 2.45
	PL	31.3 ± 3.8	32.9 ± 3.8 ^a^	0.39	0.82, 2.44
Intracellular Water (L)	B	16.9 ± 2.3	17.7 ± 2.0 ^a^	0.40	−0.10, 1.62
	PL	16.2 ± 1.9	17.3 ± 1.7 ^a^	0.55	0.57, 1.54
Extracellular Water (L)	B	15.8 ± 2.4	15.9 ± 2.4	0.05	−0.80, 0.98
	PL	15.0 ± 1.9	15.6 ± 2.1	0.29	0.08, 0.20
RF Muscle Thickness (mm)	B	2.97 ± 0.64	3.11 ± 0.67 ^a^	0.26	0.08, 0.19
	PL	2.84 ± 0.44	2.98 ± 0.54 ^a^	0.26	0.09, 0.18

*B* betaine, *PL* placebo

^a^significantly different from pre-training

^b^significantly different from placebo

### Performance

Performance data are displayed in Table 4. Vertical jump (*p* < .001, *F* = 27.97), 1 RM back squat (*p* < .001, *F* = 188.32), and 1 RM bench press (*p* < .001, *F* = 35.60) all increased over time without any differences between groups at post-testing (*p* = .869, *F* = 0.03; *p* = .275, *F* = 1.25; *p* = .254, *F* = 1.38, respectively).

**Table 4:** Performance variable outcomes

		Pre-Training	Post-Training	Effect Size	95% CI for Δ
Vertical Jump (cm)	B	40.3 ± 5.4	45.1 ± 5.5 ^a^	0.77	2.54, 6.99
	PL	39.2 ± 7.0	43.7 ± 8.4 ^a^	0.73	1.40, 7.54
Squat 1 RM (kg)	B	63.2 ± 17.9	82.9 ± 17.5 ^a^	1.20	15.40, 23.85
	PL	57.2 ± 15.2	73.9 ± 16.1 ^a^	1.02	12.61, 20.72
Bench Press 1 RM (kg)	B	36.2 ± 6.8	39.5 ± 7.8 ^a^	0.46	0.81, 5.80
	PL	33.7 ± 7.6	38.6 ± 8.6 ^a^	0.68	3.09, 6.75

*B* betaine, *PL* placebo

^a^significantly different from pre-training

^b^significantly different from placebo

### Weekly training volume and dietary intakes

Mauchly’s test of sphericity had been violated for weekly training volume (χ2(27) = 60.13, *p* = .001) and therefore the Greenhouse Geisser correction was used. Weekly training volume data is displayed in Table 5. Weekly training volume increased over time (*p* < .001, *F* = 41.38) with a trend for greater increases in the betaine group (*p* = .056, *F* = 2.38).

**Table 5:** Weekly and total training volumes

Total Weekly Volume (kg)			Effect Size
Week 1	Betaine	15,617 ± 2873	NA
	Placebo	15,192 ± 3725	NA
Week 2 ^a^	Betaine	25,259 ± 5494	2.94
	Placebo	26,540 ± 5513	3.46
Week 3 ^a^	Betaine	29,165 ± 4193	0.72
	Placebo	28,415 ± 5772	0.35
Week 4 ^a,b,c^	Betaine	34,229 ± 7774	1.02
	Placebo	29,062 ± 7296	0.13
Week 5 ^a,c,d^	Betaine	26,919 ± 5402	−0.92
	Placebo	26,367 ± 5115	− 0.34
Week 6 ^a,d^	Betaine	27,825 ± 5591	0.18
	Placebo	25,287 ± 7004	− 0.21
Week 7 ^a^	Betaine	30,283 ± 6733	0.39
	Placebo	27,129 ± 7213	0.29
Week 8 ^a,b,c,e,f^	Betaine	31,482 ± 4190	0.17
	Placebo	28,740 ± 5075	0.22
Total	Betaine	27,464 ± 4603	0.39^g^
	Placebo	25,660 ± 4593	

^a^significantly different than week 1

^b^significantly different than week 2

^c^significantly different than week 3

^d^significantly different than week 4

^e^significantly different than week 5

^f^significantly different than week 6

^g^effect size calculated as [(betaine – placebo) / pooled standard deviation]

Dietary analysis data can be found in Table 6. There were no significant differences across time (*p* > .05) or between groups at any time point (*p* > .05) for total energy, protein, carbohydrate, or fat intake.

**Table 6:** Energy and macronutrient intake

		Baseline	Post-Training
Energy (kcal/kg)	Betaine	28.1 ± 6.2	27.7 ± 7.0
	Placebo	26.2 ± 7.5	25.7 ± 6.1
Protein (g/kg)	Betaine	1.3 ± 0.4	1.3 ± 0.4
	Placebo	1.3 ± 0.3	1.1 ± 0.4
Carbohydrate (g/kg)	Betaine	4.2 ± 1.6	4.1 ± 1.4
	Placebo	3.9 ± 1.5	3.7 ± 1.5
Fat (g/kg)	Betaine	1.4 ± 0.4	1.3 ± 0.3
	Placebo	1.1 ± 0.4	1.1 ± 0.4

## Discussion

To the best of our knowledge, this was the first study to investigate the effects of chronic betaine supplementation in conjunction with supervised training on body composition and performance in females. The resistance training protocol promoted improvements in all body composition, muscle growth, and performance variables as evidenced by the significant main effects of time. The major findings of this study were that betaine supplementation enhanced body composition outcomes compared to resistance training alone, but not strength performance or rectus femoris muscle thickness.

Betaine supplementation has been shown in pigs to enhance muscular fatty acid uptake and oxidation [37] and to inhibit lipogenesis [38]. Despite substantial evidence demonstrating betaine enhances muscle growth and reduces fat mass in animals [13], research examining body composition outcomes in humans is limited [1]. In the present study betaine supplementation improved body composition by enhancing reductions in fat mass. These results are in agreement with our previous study whereby 6 weeks of betaine supplementation reduced fat mass and increased lean mass in resistance trained men [7]. In contrast, Schwab et al. [8] reported no improvements in body composition with 12 weeks of betaine supplementation in sedentary obese men and women. Discrepancies in these results may be attributed to the inclusion of exercise in our studies, whereas subjects in Schwab et al. were sedentary and instructed not to change their activity.

We also hypothesized that betaine would enhance resistance training induced increases in lean mass. While there were no statistically significant differences in increases in lean mass between groups, effect sizes slightly favored the betaine group. Differences in lean mass outcomes between the present study and our previous study [7] may be due to dietary factors. The International Society of Sports Nutrition recommends individuals engaged in resistance training consume 1.4 to 2.2 g/kg protein per day [39] and recent studies suggest that protein intakes of at least 1.8 and up to 3.1 g/kg per day may be required to offset reductions in lean mass during periods of restricted energy intake [40, 41]. Subjects in the present study consumed approximately 27 ± 6.8 kcal/kg/day, which is well below World Health Organization [42] recommendations of 35 kcal/kg/day for physically active young women. Given the caloric deficit and lower protein intake 1.3 ± 0.35 g/kg/day in the present study, it is possible that differences in lean mass may have reached significance with a higher protein intake. In partial support of this hypothesis, Lawrence et al. [43] reported a significant positive interaction between betaine supplementation and protein intake on lean mass outcomes in pigs; however, future studies are necessary to verify this hypothesis.

A second hypothesis that may explain discrepancies in lean mass outcomes between this study and our previous study in males [7] is a result of gender differences in methyl-metabolism and tissue betaine contents. Plasma betaine concentrations are under homeostatic control and are influenced by dietary betaine intake as well as by betaine-homocysteine methyltransferase (BHMT), which utilizes a methyl group from betaine to catalyze the transmethylation of homocysteine to methionine [44]. BHMT activity is suggested to play a key role in determining whether betaine is stored as tissue osmolyte or metabolized to provide methyl groups. Administration of estradiol and corticosteroids have both been shown to increase BHMT activity [45], which may explain why females typically present with lower plasma betaine and homocysteine than males [19]. To our knowledge human data regarding gender differences in betaine tissue contents are not yet available. However, Slow et al. [46] reported significant differences in tissue betaine content between genders, with female mice skeletal muscle containing approximately 42% less betaine than male mice. If more betaine is metabolized in the transmethylation of homocysteine, less plasma betaine will be available for tissue uptake. As a result, lower skeletal muscle betaine concentrations in females, despite an increased stimuli for uptake as a result of the imposed metabolic stress of exercise, may have influenced the osmotic/hypertrophic effects of betaine supplementation in the current study. Lending support to this hypothesis, the results from a pig study showed that male pigs fed diets supplemented with 1 g/kg betaine/feed more efficiently converted feed into body weight gain and had greater average reduced fat depths than females [43]; however, further research is necessary to establish differences in betaine tissue content and skeletal muscle uptake in humans.

Betaine has been shown in vitro to promote myotube differentiation and hypertrophy by increasing IGF-1 mRNA and IGF-1 proteins as well as activating the mitogen activated protein kinase (MAPK) pathways [47, 48]. In humans 2 weeks of betaine supplementation was reported to enhance Akt signaling and downstream p70 S6K phosphorylation [14]. Based on this evidence we hypothesized betaine would enhance rectus femoris muscle growth during the resistance training program. Our results do not support this hypothesis, as there were no differences between groups for increases in muscle thickness. These results are in partial agreement with our previous study where we found 6 weeks of betaine supplementation increased arm, but not leg lean cross sectional area [7]. The training program in the present study was implemented as it was previously shown to prioritize lower body hypertrophy [21]. Unfortunately, the maximal depth of the Chison Ultrasound probe is only 7.4 cm and in preliminary pilot studies this depth was not sufficient enough to measure the entire lateral quadriceps in about 33% of pilot subjects. Rather than turn potential subjects away, we decided a priori to measure only the rectus femoris muscle. It is possible that had thigh CSA or lateral quadriceps muscle thickness been measured the outcome would have been different; however, this is speculative and future research is necessary.

The effects of betaine supplementation on metrics of strength and power performance are ambiguous: a recent systemic review reported that of 7 studies published to date assessing strength and power performance, only two studies have reported positive improvements [49]. In the present study increases in vertical jump and bench press and back squat 1 RM occurred without any significant differences between groups. These results are similar to our previous study whereby betaine supplementation did not result in greater improvements in bench press or back squat 1 RM between groups [7]. In contrast, other studies that have employed isokinetic dynamometry have reported improvements in measures of power and force production with betaine supplementation [9, 11]. While 1 RM testing a valid and reliable test of force production that is specific to resistance training and sporting performance, it is possible that a familiarization effect took place over the course of the present study that may have confounded the results. Although we provided subjects with three familiarization sessions, Seo et al. [50] reported slight improvements in lower body 1 RM in female subjects over the course of 4 testing sessions.

The principal of specific adaptations of imposed demands may have also influenced the performance results. In the present study the training program was designed to optimize hypertrophy, especially in the lower body. As a result, heavy loads (> 85% 1 RM) and ballistic movements were not incorporated. Given that strength and power adaptations are maximized with heavier loads compared to higher volumes [51], future betaine training studies that include higher intensities and train muscular contractile velocity should be conducted to fully elucidate the effects of betaine supplementation on force and power output.

Since most studies that have reported improvements in performance have employed exercise tests that involved repeated higher intensity efforts [6, 20], therefore we previously hypothesized that betaine may be most ergogenic in testing and training protocols that impose a high metabolic demand [1]. Metabolic stress increases the cellular uptake of betaine which results in increased cytoplasmic osmolality, biopolymer hydration, and helps to maintain biochemical function during stress by protecting ATPase and myosin heavy-chain proteins again urea denaturation [18], attenuating reductions in the affinity of Ca^2+^ for troponin [52], and defending citrate synthase against thermodenaturation [53]. While strength-endurance was not specifically tested in the present study, a trend for greater total weekly volumes was found, with effect sizes favoring the betaine group in 4 of the 7 weeks as well as overall. These results are in agreement with our previous study, where we found betaine supplementation increased bench press work capacity in trained men only during the higher volume meso-cycles [7], and lend evidence to the hypothesis that betaine may be most ergogenic during higher volume resistance training protocols.

We hypothesized that betaine may exert ergogenic and hypertrophic effects by increasing intracellular hydration and thereby providing a more hospitable environment for excitation contraction coupling and protein synthesis. The results of the present study do not support this hypothesis, as there were no differences between groups found for total, intracellular, or extracellular water content; however, caution should be taken when interpreting the compartmental water results. First, we were unable to distinguish between compartments of intracellular water, and therefore cannot make any conclusions directly as to the effects of betaine on intramuscular hydration in particular. Second, although there were no differences between baseline and post-training nutritional status or urine specific gravity, and subjects were instructed to mimic their pre- and post-testing diets and not consume food or liquids for 3 h prior to testing, it is possible that small fluctuations in diet (sodium and fiber intake, in example) may have confounded the results. Future studies with more sophisticated equipment and strict dietary controls are required to properly assess the effects of betaine supplementation on compartmental water.

## Conclusions

In summary, the major findings of the present study are that 9 weeks of betaine supplementation improved body composition by reducing fat mass and tended to improve high-volume work capacity, but not strength or power performance in young, active, resistance training naïve females. Dietary factors, specifically suboptimal total energy and protein intake, were a limitation in the present study. While we attempted to clarify food journals to ensure accurate dietary analysis, and although under reporting of food intakes are common in the literature, subjects in the present study were likely in a caloric deficit. Despite these limitations, we can glean some practical information from the results. In particular, the results of this study suggest that betaine may be an effective fat loss supplement for females on a restricted calorie diet engaged in a resistance training program. Additionally, the trend for some enhancements in work capacity may be particularly useful to coaches working with female athletes in the aesthetic sports who commonly consume very little calories and have high training volumes in the weeks leading up to competition. Finally, given that plasma betaine is inversely associated with the loss of lean mass in middle-aged and older adults [54], further research is necessary to investigate the effects of betaine supplementation on changes in lean mass in various populations consuming a eucaloric diet.

## Abbreviations

Akt: Protein kinase B
ANOVA: Analysis of variance
BHMT: Betaine-homocysteine methyltransferase
ECW: Extracellular water
ICW: Intracellular water
LBM: Lean body mass
MAPK: Mitogen activated protein kinase
mTOR: mechanistic target of rapamycin
RM: Repetition maximum
TBW: Total body water
